# Novel Tuning of PMMA Orthopedic Bone Cement Using TBB Initiator: Effect of Bone Cement Extracts on Bioactivity of Osteoblasts and Osteoclasts

**DOI:** 10.3390/cells11243999

**Published:** 2022-12-10

**Authors:** Keiji Komatsu, Kosuke Hamajima, Ryotaro Ozawa, Hiroaki Kitajima, Takanori Matsuura, Takahiro Ogawa

**Affiliations:** 1 Weintraub Center for Reconstructive Biotechnology, School of Dentistry, University of California Los Angeles, Los Angeles, CA 90024, USA; 2 Division of Regenerative and Reconstructive Sciences, School of Dentistry, University of California Los Angeles, Los Angeles, CA 90024, USA; 3 Department of Lifetime Oral Health Care Sciences, Graduate School of Medical and Dental Sciences, Tokyo Medical and Dental University, Tokyo 113-8510, Japan

**Keywords:** arthroplasty, benzoyl peroxide, cytotoxicity, implant, poly(methyl methacrylate), tri-n-butylborane, total hip replacement

## Abstract

Bone cement containing benzoyl peroxide (BPO) as a polymerization initiator are commonly used to fix orthopedic metal implants. However, toxic complications caused by bone cement are a clinically significant problem. Poly (methyl methacrylate) tri-n-butylborane (PMMA-TBB), a newly developed material containing TBB as a polymerization initiator, was found to be more biocompatible than conventional PMMA-BPO bone cements due to reduced free radical generation during polymerization. However, free radicals might not be the only determinant of cytotoxicity. Here, we evaluated the response and functional phenotypes of cells exposed to extracts derived from different bone cements. Bone cement extracts were prepared from two commercial PMMA-BPO cements and an experimental PMMA-TBB. Rat bone marrow-derived osteoblasts and osteoclasts were cultured in a medium supplemented with bone cement extracts. More osteoblasts survived and attached to the culture dish with PMMA-TBB extract than in the culture with PMMA-BPO extracts. Osteoblast proliferation and differentiation were higher in the culture with PMMA-TBB extract. The number of TRAP-positive multinucleated cells was significantly lower in the culture with PMMA-TBB extract. There was no difference in osteoclast-related gene expression in response to different bone cement extracts. In conclusion, PMMA-TBB extract was less toxic to osteoblasts than PMMA-BPO extracts. Although extracts from the different cement types did not affect osteoclast function, PMMA-TBB extract seemed to reduce osteoclastogenesis, a possible further advantage of PMMA-TBB cement. These implied that the reduced radical generation during polymerization is not the only determinant for the improved biocompatibility of PMMA-TBB and that the post-polymerization chemical elution may also be important.

## 1. Introduction

Arthroplasty describes the partial or total surgical replacement of a defective joint with an artificial one. Artificial joint implants can be fixed with cement to bind the bone to the implant through mechanical interlocking, or alternatively cementless surgery is applied expecting implants to generate bone at the implant surface to stabilize the implant. A randomized controlled trials and the registry studies indicated that cemented implants reduce postoperative pain and provide better joint function than cementless implants, thus improving the quality of life for the patients in the areas of hip, knee, and shoulder joints [1,2,3,4,5]. However, the use of bone cement carries a risk of adverse events, with hypotension and shock widely reported in the literature [6,7]. Some complications depend on cementing techniques and are manageable; for instance, possible heat necrosis occurs by excessive cement penetration and there is a recommended protocol to prevent it. Some complications are related to the polymerization behavior and post-polymerization property of bone cement: (1) thermal stimulation during poly(methyl methacrylate) (PMMA; the main cement constituent) polymerization, (2) the local and systemic effects of monomers remained in PMMA acrylic materials, and (3) free radical generation from within bone cements during polymerization [8,9,10,11,12,13,14,15,16,17,18,19,20,21,22,23]. (Poly)methyl methacrylate-benzoyl peroxide (PMMA-BPO) bone cement, which is composed of PMMA powder, methyl methacrylate (MMA) monomer solution, and benzoyl peroxide (BPO), is widely used for orthopedic surgery [1,8,19,24,25,26,27,28]. Since all manufacturers produce bone cements with a similar chemical composition, adverse events are a persistent clinical concern regardless of the supplier [17,19,29].

There have been efforts to develop alternative bone cements that replace the BPO polymerization initiator with tri-n-butylborane (TBB) to overcome the limitations and concerns of PMMA-BPO [10,13,17,18,30]. Unlike conventional bone cements, PMMA-TBB materials have several biological benefits including reduced cytotoxicity and enhanced proliferation and differentiation of osteoblasts [10,13,17,18]. Despite growing evidence of the benefits of PMMA-TBB, the underlying cytochemical mechanisms responsible for its biocompatibility remain poorly understood, although reduced generation of heat and free radicals from the material during polymerization has been suggested as putative mechanisms [10,17,18]. The effects of other components potentially released from the material remain unclear. In light of long-lasting superior biocompatibility of PMMA-TBB over PMMA-BPO cements reported recently [17], its potential advantage in terms of post-polymerization chemistry would be of particular interest.

Aseptic loosening of cemented arthroplasty joints caused by local osteolysis at the cement–bone interface is also a severe orthopedic problem [31,32,33]. Many studies have reported that the use of PMMA-BPO bone cement is associated with early and continuous bone resorption due to enhanced osteoclast proliferation, suggesting that active osteoclasts may be responsible for aseptic loosening of joints [34,35,36,37]. PMMA-based bone cement is also known to stimulate the release of bone resorption-inducing and proinflammatory factors such as prostaglandins (PGs), interleukin 1 (IL-1), IL-6, and tumor necrosis factor (TNF) from macrophages [38,39]. Materials that prevent or mitigate osteoclast proliferation and activation would be desirable. In particular, the effect of PMMA-TBB on osteoclasts remains to be tested.

In order to explore the mechanism underlying the excellent biocompatibility and an additional biological advantage of PMMA-TBB, we assessed the effects of bone cement extracts derived from an experimental PMMA-TBB and two commercially available PMMA-BPO cements on the response and function of bone marrow-derived osteoblasts and osteoclasts.

## 2. Materials and Methods

### 2.1. Preparation of Bone Cement Extracts

The composition of the three different bone cements used in this study are detailed in Table 1. As reported previously [10,18], PMMA-BPO1 bone cement was prepared by mixing powder and liquid in the recommended ratio (powder (wt): liquid (vol) ratio = 40:20; Surgical Simplex P, Stryker, Kalamazoo, MI, USA). PMMA-BPO2 bone cement was prepared by mixing the powder and liquid in the recommended ratio (powder (wt): liquid (wt) ratio = 40:18.88; Endurance, DePuy Orthopaedics, Warsaw, IN, USA). For PMMA-TBB resin, TBB initiator was added to the MMA monomer in a ratio of 9% to make a liquid mix. Then, the PMMA powder and liquid were mixed in the ratio (*wt*/*wt*) 40:18.8. The MMA, PMMA, and TBB materials were manufactured and provided by Mitsui Chemical Inc. (Tokyo, Japan). All bone cement materials were prepared using the same protocol, specifically, by mixing for 30 s at room temperature of 25 °C with 55% humidity. The mixing was performed under a non-vacuum by a hand-spatula in a hemispherical porcelain basin (20 mm diameter and 10 mm depth). As established previously [24], 1 g of the mixed bone cement was placed at the bottom of 50 mL conical tubes (BD Biosciences, Franklin Lakes, NJ, USA), allowing polymerization for 15 min, adding 5 mL of α-modified Eagle’s medium (α-MEM; Gibco BRL Division of Invitrogen, Gaithersburg, MD, USA) containing serum and antibiotics, and incubating for 72 h at 37 °C. These solutions were used as bone cement extracts in subsequent experiments.

### 2.2. Rat Femur-Derived Osteoblasts Culture

Primary osteoblasts were cultured from rat bone marrow stromal stem cells as previously reported [40]. Femurs were excised aseptically from 8-week-old male Sprague Dawley rats. Soft tissues and muscles were removed and washed with 1× phosphate-buffered saline (PBS; Gibco, Carlsbad, CA, USA) several times. Both ends of the femur bone were carefully severed and the bone marrow was flushed out using 3 mL of an osteoblastic-differentiating medium through a 20-gauge needle. The osteoblastic-differentiating medium contained a-modified Eagle’s medium, 15% fetal bovine serum (FBS), 50 μg/mL ascorbic acid, 10^−8^ M dexamethasone, 10 mM Na-ß-glycerophosphate, and antibiotic-antimycotic solution containing 10,000 units/mL penicillin G sodium, 10,000 mg/mL streptomycin sulfate, and 25 mg/mL amphotericin B. The extracted cells were cultured in 100 mm dishes, and at 80% confluency, the cells were detached using 0.25% trypsin 1 mM EDTA 4Na and passaged. After 3-time passaging, the cells were seeded into each well of 12-well culture-grade polystyrene plates at a density of 1.0 × 10^4^ cells/cm^2^. As determined previously [24], 100 µL of bone cement extract prepared, as described above, was added to each well. Three wells were prepared for each cement group per experiment (*n* = 3). Culture medium containing the extracts was renewed every 3 days. All experiments were performed following protocols approved by The Chancellor’s Animal Research Committee at the University of California at Los Angeles (ARC #2005-175-41E, approved 30 January 2018), the PHS Policy for the Humane Care and Use of Laboratory Animals, and the UCLA Animal Care and Use Training Manual guidelines.

### 2.3. Cell Quantification

The number of cells attached was evaluated after one day of culture. After aspirating the culture medium, cells were gently washed with PBS, and 0.25% trypsin 1 mM EDTA 4 Na was added and incubated at 37 °C for 5 min to completely detach the remaining cells in the wells. Cells were counted using a hematocytometer.

### 2.4. Morphology and Spreading Behavior of Osteoblasts

The cell morphology and cytoskeletal arrangement were visualized and evaluated by fluorescence microscopy (DMI 4000 B, Leica Microsystems Inc., Buffalo Grove, IL, USA) and confocal laser scanning microscopy (TCS SP5, Leica, Wetzlar, Germany). On day 1 and 3 of culture, osteoblasts were fixed with 10% formalin and stained with 4′,6-diamidino-2-phenylindole (DAPI, Vector Laboratories, Burlingame, CA, USA) for nuclei, rhodamine phalloidin for actin filaments (Molecular Probes, Eugene, OR, USA), and antibodies targeting vinculin (ab11194, Abcam, Cambridge, UK). The area, perimeter, and Feret’s diameter of single cells were measured using ImageJ (ver.1.51j8, NIH, Bethesda, MD, USA) for quantitative cytomorphometric analysis.

### 2.5. Cell Proliferation

To evaluate cellular proliferation, the density of propagated cells after 3 and 5 days of culture was evaluated with a hematocytometer, as described above. The proliferative activity of osteoblasts was also confirmed by 5-bromo-2′-deoxyuridine (BrdU) incorporation during DNA synthesis. At days 3 and 5 of culture, 100 mL of 100 mM BrdU solution (Roche Applied Science, Mannheim, Germany) was added to the culture wells followed by incubation for 10 h. After trypsinizing the cells and denaturing the DNA, cultures were incubated with peroxidase-conjugated anti-BrdU for 90 min and reacted with tetramethylbenzidine for color development. Absorbance at 370 nm was measured using an ELISA plate reader (Synergy HT, BioTek Instruments, Winooski, VT, USA).

### 2.6. Alkaline Phosphatase (ALP) Activity

Osteoblast ALP activity was examined on days 3 and 7 using colorimetry-based and histochemical-based assays. The colorimetry-based assay was performed as follows. Cultured cells were rinsed with double-distilled water and treated with 250 μL p-nitrophenyl phosphate (LabAssay ALP, Wako Pure Chemicals, Richmond, VA, USA) and further incubated at 37 °C for 15 min. ALP activity based on colorimetry was evaluated as the amount of nitrophenol released through the enzymatic reaction and determined at 405 nm using an ELISA plate reader (Synergy HT). The histochemical-based assay was performed as follows. Cultured cells were washed twice with PBS and incubated with 120 mM Tris buffer (pH 8.4) containing 0.9 mM naphthol AS-MX phosphate and 1.8 mM fast red TR at 37 °C, 30 min.

### 2.7. Rat Femur-Derived Osteoclasts Cell Culture

As reported elsewhere [41], osteoclasts were induced from bone marrow cells. Bone marrow cells were extracted from the femurs of 8-week-old male Sprague Dawley rats similarly using the method described above and placed in α-MEM containing 10% FBS and antibiotics (100 U/mL penicillin, 100 μg/mL streptomycin; Wako Pure Chemical Industries, Ltd., Osaka, Japan). The cells were collected as a pellet by centrifuging and then seeded into 12-well plates at densities of 1.0 × 10^4^ cells/cm^2^. After two days, the culture medium was renewed and supplemented with 10 ng/mL macrophage colony-stimulating factor (M-CSF; PeproTech, Inc., Rocky Hill, NJ, USA). After a 2-day incubation, the culture medium was changed to one containing 10 ng/mL M-CSF, and 50 ng/mL receptor activator of nuclear factor κB ligand (RANKL) (PeproTech, Inc.), and finally added with 100 µL/well of bone cement extract.

### 2.8. Tartrate-Resistant Acid Phosphatase (TRAP) Staining

After 60 h exposure to bone cement extracts, osteoclasts were fixed in 10% neutral buffered formalin at room temperature and stained with TRAP for 1 h at 37 °C. The number of TRAP-positive cells containing three or more nuclei was determined. All positive cells in each well were counted using a light microscope.

### 2.9. Real-Time Quantitative (q)PCR

Gene expression was analyzed by qPCR on days 1 and 4 after exposure to bone cement extracts. Total RNA was extracted from cells using TRIzol (Invitrogen, Carlsbad, CA, USA) and a Direct-zol RNA MiniPrep kit (Zymo Research, Irvine, CA, USA). Extracted RNA was reverse transcribed into first-strand cDNA using SuperScript III Reverse Transcriptase (Invitrogen, Carlsbad, CA, USA). qPCR was performed in a 20 μL volume containing 90 ng cDNA, 10 μL TaqMan Universal Master Mix II, and 1 μL TaqMan Gene Expression Assay using the QuantStudio3 Real-Time PCR System (Thermo Fisher Scientific, Waltham, MA, USA) to quantify the expression of nuclear factor of activated T cells cytoplasmic 1 (NFATc1), TRAP, and cathepsin K. Gapdh expression was used as the endogenous control.

### 2.10. Statistical Analysis

All cell culture experiments were conducted in triplicate (*n* = 3). One-way ANOVA was used to determine differences of each variable among three different bone cement materials using GraphPad (Software, Inc., San Diego, CA, USA). If the ANOVA result was significant, the Bonferroni post hoc test was used for multiple comparisons within the three bone cements after confirming the normal distribution; <0.05 was considered statistically significant.

## 3. Results

### 3.1. Improved Attachment of Osteoblasts with PMMA-TBB Extract

**Figure 1:**
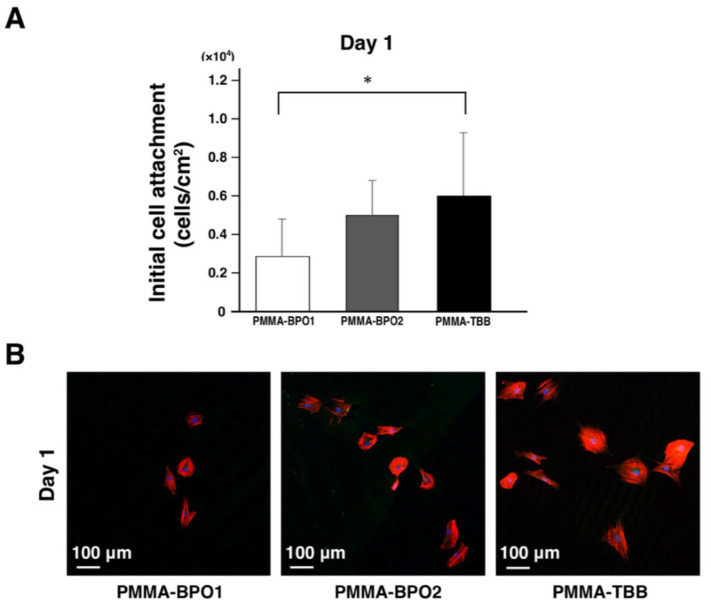
Initial osteoblast attachment to culture wells after 24 h of culture with three different bone cement extracts. (**A**) The number of cells attached to the culture wells after one day of culture was evaluated by manually counting cells using a hemocytometer. Data shown are mean ± SD (*n* = 3). Significant differences between the three groups are shown (one-way ANOVA followed by Bonferroni correction, * *p* < 0.05. (**B**) Representative fluorescence microscopy images of initially attaching osteoblasts after 24 h of culture stained with rhodamine phalloidin for actin filaments (red) and 4′,6-diamidino-2-phenylindole (DAPI) for nuclei (blue) at low magnification.

We first evaluated the number of osteoblasts settling and attaching to the wells after 24 h of culture with bone cement extracts (Figure 1A). Significantly more osteoblasts attached to the wells in the culture with PMMA-TBB extract than in the culture with PMMA-BPO1. Low-magnification fluorescence microscopy at 24 h after culture showed the presence of osteoblasts in all cultures but there were more cells in the culture with PMMA-TBB extract than with PMMA-BPO extracts, confirming the above result (Figure 1B).

### 3.2. Improved Proliferation of in Culture with PMMA-TBB Extract

**Figure 2:**
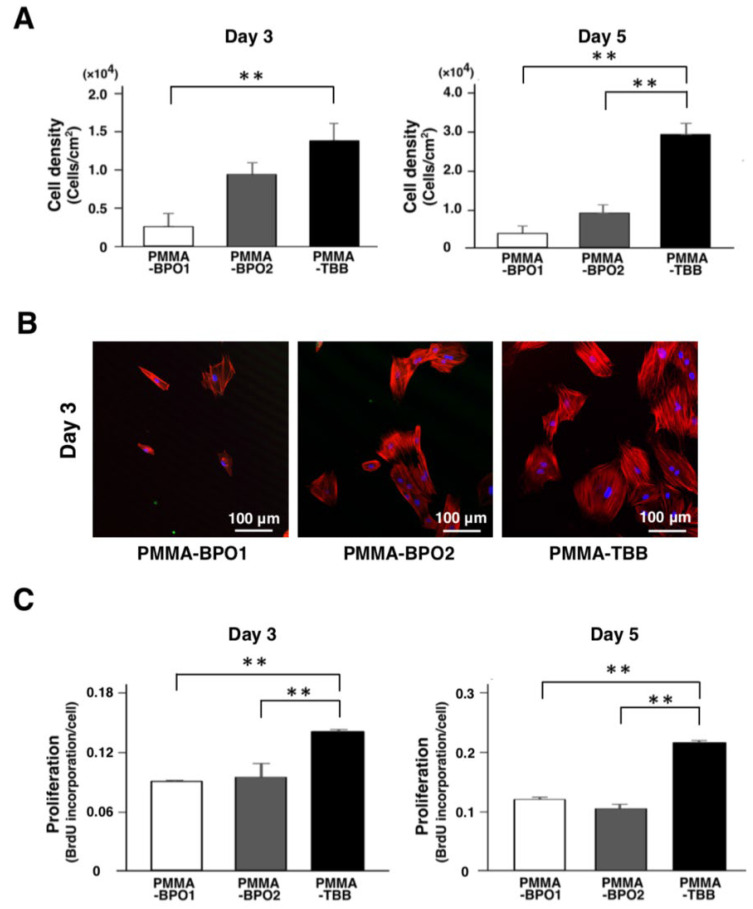
Evaluation of the proliferation of osteoblasts exposed to three different bone cement extracts. (**A**) The density of propagated cells after 3 and 5 days of culture was evaluated by manually counting cells using a hemocytometer. (**B**) Representative fluorescence microscopy images of propagated osteoblasts after 3 days of culture stained with rhodamine phalloidin for actin filaments (red) and 4′,6-diamidino-2-phenylindole (DAPI) for nuclei (blue) at low magnification. (**C**) BrdU incorporation per cell measured at days 3 and 5. Data shown are mean ± SD (*n* = 3). Significant differences between the three groups are shown (one-way ANOVA followed by Bonferroni correction, ** *p* < 0.01).

To evaluate osteoblast proliferation, the density of propagated cells was evaluated on days 3 and 5 of culture. It was significantly higher with PMMA-TBB extract than with PMMA-BPO1 extract on day 3 (Figure 2A). On day 5, the cell density with the PMMA-TBB extract was higher than that with both PMMA-BPO extracts. Fluorescence microscopy images on day 3 confirmed that the number of propagated cells exposed to PMMA-TBB bone cement extract was considerably higher than that of the two PMMA-BPO bone cement extract groups (Figure 2B). BrdU incorporation per cell on days 3 and 5 was also higher with the PMMA-TBB extract than with the two PMMA-BPO extracts, confirming the results of cell density (Figure 2C).

### 3.3. Improved Spreading Behavior of Osteoblasts with PMMA-TBB Extract

**Figure 3:**
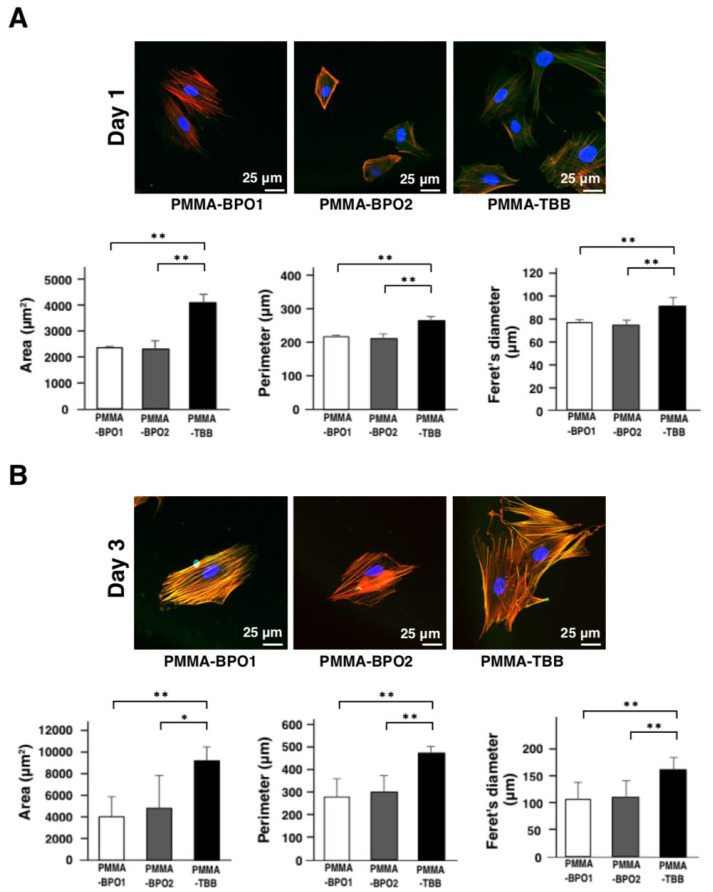
Spreading and cytoskeletal arrangement of osteoblasts exposed to three different bone cement extracts 1 and 3 days after seeding. Representative high magnification confocal microscopy images of the spreading behavior of osteoblasts stained with rhodamine phalloidin for actin filaments (red), DAPI for nuclei (blue), and vinculin (green) after (**A**) 1 day and (**B**) 3 days of culture (top panels). Cell morphometry analysis of the images (histograms shown below). Data shown are mean ± SD (*n* = 5). Significant differences between the three groups are shown (one-way ANOVA followed by Bonferroni correction, * *p* < 0.05, ** *p* < 0.01).

The spreading behavior of osteoblasts was evaluated qualitatively and quantitatively on days 1 and 3 by confocal laser microscopy. At both time points, there was significant more spreading and cytoskeletal development in PMMA-TBB extract-exposed osteoblasts than PMMA-BPO extract-exposed osteoblasts (Figure 3A,B). In particular, osteoblasts with PMMA-TBB extract showed enhanced development of discernible cytoplasmic projections and cytoskeletal actin expression on day 3 of culture. Furthermore, the expression of the focal adhesion protein vinculin was more intense in osteoblasts with PMMA-TBB extract than those with other bone cement extracts. Quantitative cytomorphometry showed that the area, perimeter, and Feret’s diameter of PMMA-TBB extract-exposed osteoblasts were significantly higher than for PMMA-BPO extract-exposed osteoblasts on both days 1 and 3 of culture (Figure 3A,B).

### 3.4. Enhanced Osteogenic Differentiation in Osteoblasts with PMMA-TBB Extract

**Figure 4:**
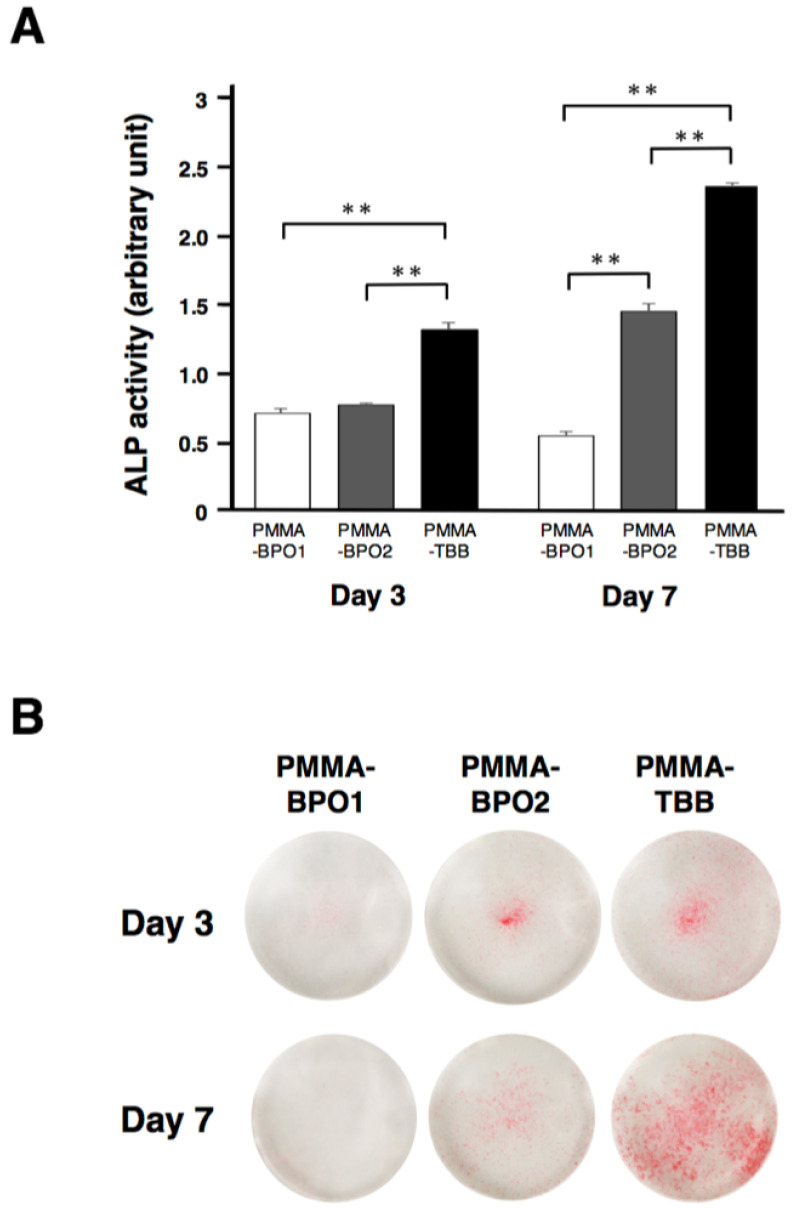
Differentiation of osteoblasts exposed to three different bone cement extracts. Alkaline phosphatase (ALP) evaluation of osteoblasts exposed to three different bone cement extracts 3 and 7 days after seeding. (**A**) Colorimetric quantification of ALP activity. (**B**) Representative images of ALP staining. Data shown are mean ± SD (*n* = 3). Significant differences between the three groups are shown (one-way ANOVA followed by Bonferroni correction, ** *p* < 0.01).

The alkaline phosphatase (ALP) activity of osteoblast cultures was measured on day 3 and day 7. Quantitative analysis showed a significant increase in ALP activity in osteoblasts with PMMA-TBB extract compared to osteoblasts with PMMA-BPO extracts on both days. The ALP activity of PMMA-BPO1 culture decreased from day 3 to 7 (Figure 4A). The results of ALP staining confirmed the quantitative results. (Figure 4B).

### 3.5. Reduced Osteoclastogenesis with PMMA-TBB Extract

**Figure 5:**
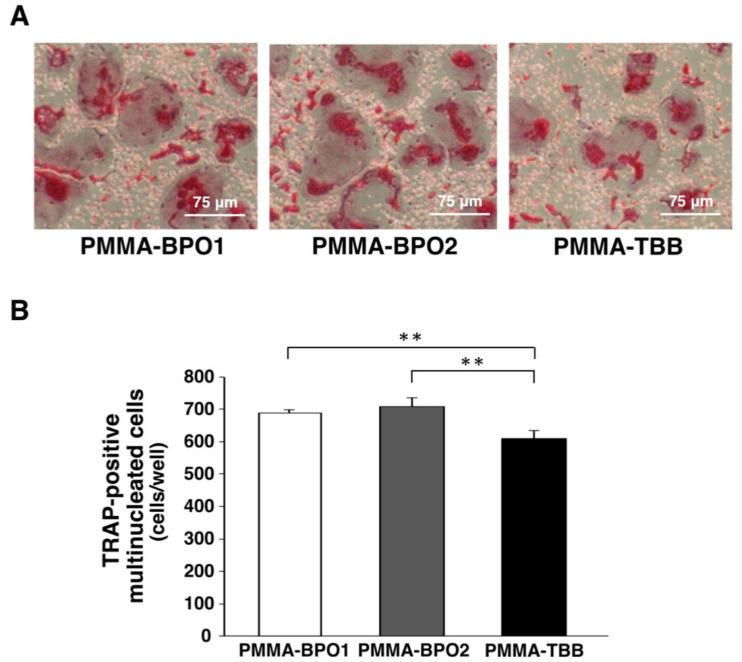
Evaluation of TRAP activity of osteoclasts after 3 days of bone cement extract exposure. (**A**) Representative images of TRAP staining. (**B**) The number of TRAP-positive multinucleated cells as determined by the number of cells containing 3 or more nuclei. Data shown are mean ± SD (*n* = 3). Significant differences between the three groups are shown (one-way ANOVA followed by Bonferroni correction, ** *p* < 0.01).

We next performed TRAP staining of osteoclasts on day 3 after exposure to bone cement extracts. TRAP-positive multinuclear cells were detected in all samples after 3 days of exposure. The number of TRAP-positive cells was similar in the two PMMA-BPO extract-exposed groups but there were significantly fewer osteoclasts in the PMMA-TBB extract-exposed group. Osteoclasts appeared smaller with PMMA-TBB extract (Figure 5A,B).

### 3.6. Evaluation of Osteoclast Function

**Figure 6:**
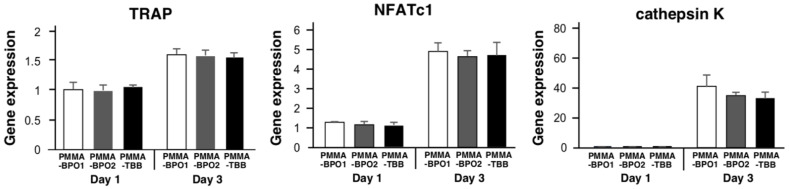
Evaluation of osteoclast activity stimulated by three different bone cement extracts. Relative gene expression levels of osteoclast TRAP, NFATc1, and cathepsin K after 1 and 3 days of exposure to bone cement extracts. Data shown are mean ± SD (*n* = 3).

The expression of osteoclast-related genes by cells exposed to different cement extracts was examined by qPCR on days 1 and 3 of exposure. The expression increased from day 1 to 3 for all three genes tested in all bone cement groups (Figure 6). The expression of all genes showed no significant difference among different bone cement extracts.

## 4. Discussion

Previous in vitro studies investigating the behavior and responses of cells to PMMA-TBB materials only examined cells grown on the material. Here, we took a different approach, instead exposing cells to the material extracts to simulate solely the exposure of cells to the various chemicals after polymerization. Since material-derived substances are continuously eluted into the local environment in situ, it is important from the clinical perspective to investigate their cytotoxicity not only to cells in contact with the material but also to cells and tissues in proximity and/or distant from the material. Our results clearly showed that even though the cells were not in direct contact with the bone cement, they were affected by dissolved constituents produced by the bone cement. Furthermore, PMMA-TBB bone cement has been demonstrated to be superior to conventional PMMA-BPO bone cements even in experiments focusing on the post-polymerization effects.

The number of osteoblasts initially attaching to the culture wells incubated with bone cement extract tended to be higher for PMMA-TBB than PMMA-BPO cements. These data suggest that BPO interfered with osteoblast settlement, attachment, and proliferation more than TBB, especially with respect to proliferation based on the more pronounced result of cell density on day 5 than on day 3. Previous reports showed 15–20-times higher free radical generation from PMMA-BPO bone cement compared with PMMA-TBB bone cement 24 h after cement mixing, suggesting that PMMA-BPO’s toxicity to osteoblasts can be explained by the abundant generation of free radicals [10]. The polymerization radicals indeed cause a strong oxidative stress on cultured cells, and their adverse biological effects were demonstrated in several scavenging experiments using antioxidants [9,10,11,19,22,23,24,42,43]. However, free radicals are highly reactive, short-lived, and unstable molecules, so they are unlikely to be present in extracts [44]. Therefore, it is reasonable to assume that the adverse biological effects on cells in this study were caused by bone cement components other than free radicals. On the other hand, it is known that residual monomers are highly detectable in the extracts of PMMA-based materials and highly toxic to cause post-operation complications [19,23,45,46,47,48,49]. The PMMA-TBB material used in this study may have produced a less residual monomer than PMMA-BPO cements.

The critical difference in chemical composition between PMMA-TBB and PMMA-BPO materials is the polymerization initiator. Resin materials containing TBB as a polymerization initiator are widely used as cementing materials in dentistry [18,50,51,52,53], because of the higher degree of polymerization [54]. Another unique feature of TBB-containing cements is that the polymerization reaction proceeds effectively in a humidified environment [17,55]. We collected bone cement extract by immersing cement mixtures in culture media for three days and waiting for the release of chemical components from the polymerized material. In these wet conditions, the PMMA-TBB bone cement may have polymerized more completely than PMMA-BPO, resulting in a less residual monomer and other chemicals. Since bone cement is placed in the hip or knee joint along with orthopedic metal implants and remains in the body semi-permanently, a bone cement with a TBB initiator that is less cytotoxic in wet environments may be more advantageous than conventional PMMA-BPO bone cements.

BPO has also been reported to be toxic at the molecular level, specifically, via genotoxicity and thereby the reduced differentiation of osteoblasts and induced apoptosis [56,57], and we hypothesized that PMMA-TBB would not be as toxic as its conventional BPO-containing counterparts. ALP activity, a marker of osteoblast differentiation [58,59,60], was significantly higher in cells exposed to PMMA-TBB than PMMA-BPO, which supported our hypothesis. Further, there may be a harmful effect by BPO remnant after polymerization. The BPO remnant may permeate into cells, react with intracellular substances, and create radicals [61]. Such endogenous oxidative stress may jeopardize viability and function of the cells. Another important discussion would be that several studies have reported that boron, a degradation product of TBB, promotes osteoblast differentiation [62,63,64,65]. Quantification of boron in the extracts and the identification of the role of the TBB bone cement-derived boron in osteoblast differentiation would be of particular importance in future studies to understand the mechanism behind the better biocompatibility of PMMA-TBB materials.

To the best of our knowledge, this is the first study examining the effect PMMA-TBB materials on osteoclasts. Previous studies have shown that several proinflammatory cytokines such as TNF and IL-1 are induced by PMMA debris/particles generated after cemented arthroplasty, promoting osteoclastogenesis [66,67,68]. We hypothesized that different types of bone cement will have different effects on osteoclastogenesis due to the different chemical components released by different bone cements. Although there was no difference in the expression of osteoclast markers such as cathepsin K, NFATC1, and TRAP, the number of TRAP-positive multinucleated cells was significantly lower after the exposure to PMMA-TBB extract than to conventional bone cement extracts. This is a new finding that uncovered that bone cement elution alone without a physical debris/particle may influence the local environment to induce bone resorption and support the PMMA-TBB as an alternative bone cement.

BPO decomposes in a reaction with the polymerization accelerator N,N-dimethyl p-toluidine (DmpT) to produce two radicals that then combine to form a phenyl radical, which functions as an activated polymerization initiator. DmpT is known to be cytotoxic [69,70,71]. In contrast, the TBB polymerization initiator does not require DmpT and reacts with oxygen to generate radicals and initiate polymerization. In addition to the presence or absence of toxic DmpT, the difference in polymerization mechanisms between BPO- and TBB-based materials may determine the level of cytotoxicity. Future studies should address which particular chemicals eluted from the materials are responsible for cytotoxicity.

A question remains unanswered on the dose-dependent effect of BPO. PMMA-BPO1 with 1.3% BPO was more cytotoxic than PMMA-BPO2 with 1.85% BPO, as shown in the results of cell attachment on day 1, cell dentistry on days 3 and 5, and ALP activity on day 7, implying that BPO dose alone does not determine the material toxicity. In fact, PMMA-BPO1 contains a greater percentage of DmpT, a source of toxicity, as mentioned above, which may explain, at least partially, the higher toxicity of PMMA-BPO1. Another possibility would be the residual monomer due to the insufficient BPO in the PMMA-BPO1, which creates another source of toxicity. This study revealed the toxicity levels of three different bone cements as a holistic effect of eluted chemicals. Future studies should address the role of each chemical ingredient and/or elution in determining the biocompatibility and also how to optimize them. A particular interest is also to culture cells directly on the materials, which may reflect the effect of real-time polymerization behaviors such as time-dependent changes of temperature, chemical release, and free radical production.

After knowing the higher biocompatibility of the PMMA-TBB experimental material, the evaluation on its various mechanical properties will be necessary. International Standard ISO 5833:2002 (Implants for surgery—Acrylic resin cements) specifies the physical and mechanical requirements for curing polymerizing resin cement. It is crucial to determine whether the higher biocompatibility of PMMA-TBB concurrently meets the requirements as a load-bearing device. The high compatibility of PMMA-TBB may hypothesize a higher degree of completion in polymerization. If the polymerization completes similarly to or more maturely than the PMMA-BPO bone cements used in this study, satisfactory results of mechanical testing may be anticipated.

We designed the experimental protocol considering the polymerization behavior, working time, and workability of the materials. Each bone cement material has a recommended mixing and setting protocol by the manufacturers, established on the products. We were required to use the materials at a substantially smaller scale than the products for the experimental purpose. Fortunately, a hand-mixing protocol for 30 s was feasible for all cements used without hardening in the middle. To allow sufficient and equal setting time among different materials, we waited for 15 min before adding the culture medium. All materials showed a similar transition from doughy to hardening stage with this protocol. However, we need to take into consideration that this protocol was particular to this study and different from the clinical protocols, in terms of multiple factors such as the material volume, absence/presence of vacuum, and modification from the product-specific instruction. In particular, as described above, the newly introduced PMMA-TBB uses a unique mechanism of triggering polymerization. Various material properties of this material during and after polymerization requires further studies to determine clinical workability and suitability as a load-bearing material. From this context, the choice of bone cement extract, instead of culturing cells directly on the materials, may have been advantageous. Incubation time for 72 h to produce the extract may have contributed to the standardization or minimization of the factors arising from the different polymerization behaviors of different cement materials. In this bone cement extract model, we chose not to include the untreated cells. Due to the considerable difference in toxicity between untreated cells and treated cells, optimizing the assay protocols was extremely challenging. Therefore, we focused on the comparison among the three different bone cements. In the future, the comparison between the untreated and treated cells may be possible by using more diluted bone cement extracts with mitigated toxicity.

## 5. Conclusions

Chemicals eluted from two PMMA-BPO bone cements were more toxic to osteoblasts than the experimental PMMA-TBB material. Osteoclastogenesis was reduced in the culture with chemicals eluted from PMMA-TBB than those from PMMA-BPO bone cements, which may be a novel advantage of PMMA-TBB materials to be explored as an alternative bone cement material. Post-polymerization chemistry of bone cements is a crucial factor to determine their biocompatibility.

## Figures and Tables

**Table 1:** The constituents of three different bone cement materials were used in this study.

Bone Cement Material	Constituents
PMMA-BPO1 (Surgical Simplex P, Stryker, Kalamazoo, MI, USA)	Powder: Polymethyl methacrylate (15.00%) Methyl methacrylate-styrene copolymer (73.70%) Benzoyl peroxide initiator (1.30%) Barium sulfate (10.00%) Liquid: Methyl methacrylate monomer (97.40%) N,N-dimethyl p-toluidine (2.60%) Hydroquinone (75 ppm)
PMMA-BPO2 (Endurance MV, DePuy Synthes, Warsaw, IN, USA)	Powder: Polymethyl methacrylate (67.05%) Methyl methacrylate/styrene copolymer (21.10%) Benzoyl peroxide initiator (1.85%) Barium sulfate (10.00%) Liquid: Methyl methacrylate (98.0%) N,N-dimethyl-p-toluidine (<2.00%) Hydroquinone (75 ppm)
PMMA-TBB (Experimental)	Powder: Polymethyl methacrylate (90.00%) Barium sulfate (10.00%) Liquid: Methyl methacrylate (91.00%) Tri-n-butyl borane initiator (9.00%) Hydroquinone (50 ppm)

## Data Availability

Data availability on request from the authors.
